# Performance of LED-Based Fluorescence Microscopy to Diagnose Tuberculosis in a Peripheral Health Centre in Nairobi

**DOI:** 10.1371/journal.pone.0017214

**Published:** 2011-02-18

**Authors:** Maryline Bonnet, Laramie Gagnidze, Willie Githui, Philippe Jean Guérin, Laurence Bonte, Francis Varaine, Andrew Ramsay

**Affiliations:** 1 Epicentre, Paris, France; 2 Centre for Respiratory Diseases Research, Kenya Medical Research Institute, Nairobi, Kenya; 3 Médecins Sans Frontières, Paris, France; 4 UNICEF/UNDP/World Bank/WHO Special Programme for Research and Training in Tropical Diseases, World Health Organization, Geneva, Switzerland; Hopital Raymond Poincare - Universite Versailles St. Quentin, France

## Abstract

**Background:**

Sputum microscopy is the only tuberculosis (TB) diagnostic available at peripheral levels of care in resource limited countries. Its sensitivity is low, particularly in high HIV prevalence settings. Fluorescence microscopy (FM) can improve performance of microscopy and with the new light emitting diode (LED) technologies could be appropriate for peripheral settings. The study aimed to compare the performance of LED-FM versus Ziehl-Neelsen (ZN) microscopy and to assess feasibility of LED-FM at a low level of care in a high HIV prevalence country.

**Methods:**

A prospective study was conducted in an urban health clinic in Nairobi, Kenya. Three sputum specimens were collected over 2 days from suspected TB patients. Each sample was processed with Auramine O and ZN methods and a 4^th^ specimen was collected for TB culture reference standard. Auramine smears were read using the same microscope, equipped with the FluoLED™ fluorescence illuminator. Inter-reader agreement, reading time and technicians' acceptability assessed feasibility.

**Results:**

497 patients were included and 1394 specimens were collected. The detection yields of LED-FM and ZN microscopy were 20.3% and 20.6% (p = 0.64), respectively. Sensitivity was 73.2% for LED-FM and 72% for ZN microscopy, p = 0.32. It was 96.7% and 95.9% for specificity, p = 0.53. Inter-reader agreement was high (kappa = 0.9). Mean reading time was three times faster than ZN microscopy with very good acceptance by technicians.

**Conclusions:**

Although it did not increase sensitivity, the faster reading time combined with very good acceptance and ease of use supports the introduction of LED-FM at the peripheral laboratory level of high TB and HIV burden countries.

## Introduction

Tuberculosis (TB) remains a global public health emergency which disproportionately affects the poorer countries of the world [1]. In these countries with high TB burdens the laboratory infrastructure for the diagnosis of infectious diseases is not adequately resourced. The only diagnostic technique for TB suitable to peripheral levels of health services is serial sputum smear microscopy. Sputum smear microscopy is associated with low and variable sensitivity, exacerbated in high HIV prevalence settings [2]. Sensitivity is largely determined by the duration of microscopic examination. Where workloads are high and the amount of time spent examining smears is low, sensitivity is correspondingly low [3]. Laboratory infrastructure development is urgently needed, as is the development of more sensitive and rapid TB diagnostics more suitable for peripheral settings. Unfortunately, adequate improvement in neither field can be expected in the short term [4], [5]. Recognizing this, a number of research groups have aimed to improve the performance of smear microscopy through new technology and service delivery approaches.

A series of systematic reviews and additional research have led to WHO recommendations aimed at improving the efficiency of smear microscopy services [6]–[11]. These recommendations have included the adoption of more sensitive definitions of positive smears and smear positive cases, as well as reducing the number of sputum specimens to be examined [12]. A systematic review published in 2006 reported that fluorescence microscopy (FM) could improve sensitivity of smear microscopy by 10% over the conventional Ziehl-Neelsen (ZN) microscopy mostly used in poorer countries, and that specificities of the two techniques were comparable [7]. It also reported findings of one large study from the 1960s demonstrating that the performance of ZN microscopy service is comparable to that of an FM service in which smears are examined for 25% of the time taken to examine ZN smears [13]. Until recently the implementation of FM was problematic due to the complexity of the equipment and dependence upon a steady electrical supply and ultra-violet light generated by expensive mercury vapour lamps with short life-spans [7]. However, the advent of simple FM systems based on light-emitting diodes (LED-FM) which have long life-spans, do not produce UV light, and have minimal power requirements could facilitate the implementation of FM in high burden and limited resources countries [14]. A number of LED-FM microscopes or adaptors that can convert existing light microscopes to LED-FM, have been evaluated and/or are on the market [15]–[22].

This study aimed to assess the accuracy of LED-FM for the diagnosis of TB compared to ZN microscopy in a high HIV-prevalence country at a peripheral health centre. The diagnostic accuracy was assessed in the context of the various WHO efficiency recommendations described above.

## Methods

### Ethics Statement

The Ethical Review Committees of KEMRI (Nairobi, Kenya) and the “Comité de Protection des Personnes” (Saint Germain en Laye, France) approved the study. Patients signed informed consent form to participate in the study.

**Figure 1:**
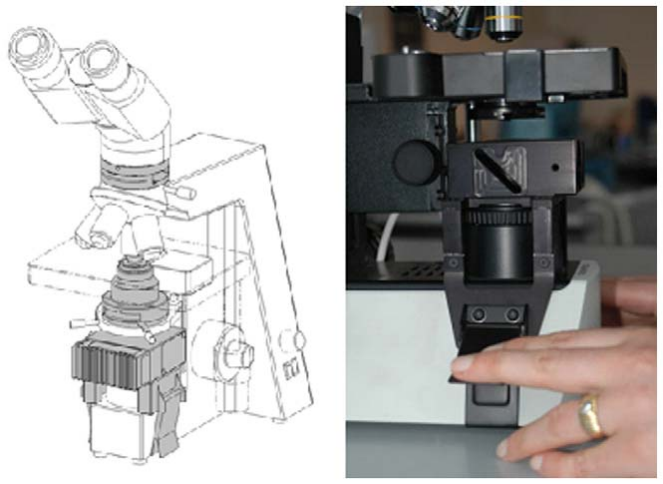
LED Fluoled fluorescence illuminator® (*Fraen Corporation Srl, Italy*). Source: http://www.fraensrl.com.

The study was conducted in an urban HIV/TB clinic, supported by Médecins Sans Frontières in Mathare, a slum area of Nairobi, Kenya. The clinic's laboratory routinely performs direct sputum ZN microscopy. All consecutive patients over 14 years of age with a cough for more than 2 weeks were eligible for the study. After giving written informed consent, patients submitted three sputum specimens over two consecutive days. Patients received instructions on production of good quality specimen [23]. The 1^st^ specimen was collected on the spot at initial consultation; the 2^nd^ in the early morning at home and the 3^rd^ on the spot when patient delivered the morning specimen to the clinic. Two smears were prepared per specimen. One was stained with the hot ZN method using carbol-fuchsin 1% and the other with the auramine O for fluorescence microscopy. Auramine smears were counterstained with potassium permanganate for 60 seconds. Attribution of staining method per smear was randomized. ZN slides were examined by bright-field microscopy (magnification, X 1,000) on CX21 Olympus microscope. Auramine slides were read using the same microscope, equipped with the LED Fluoled fluorescence illuminator® (*Fraen Corporation Srl, Italy*) (magnification, X400). No dark room was used for LED-FM. The number of acid fast bacilli (AFB) read per standard length of 2 cm long and 1 cm large was reported. A length corresponded to 100 fields under 1000 magnification and was estimated to be equivalent of 20 fields under 400 magnification [24]. Slides were read by two independent laboratory technicians who were blind to the LED-FM smears result when reading the ZN smears from same specimen and vice versa. Each smear result was independently recorded on a separate laboratory form. Staff rotation prevented the same technician from reading all smears from the same patient. Table 1 presents the AFB grading reading scales used during the study for the ZN and LED-FM, respectively. Figure 1 shows the FluoLED system.

**Table 1:** Smear microscopy grading scales [23].

Result	BRIGHTFIELD1000× magnification1 length = 100 HPF	FLUORESCENCE A400× magnification1length = 40 fields = 200 HPF	FLUORESCENCE B200× magnification1 length = 30 fields = 300 HPF
Negative	Zero AFB/1 length	Zero AFB/1 length	Zero AFB/1 length
Scanty	1–9 AFB/1 length or 100 HPF	1–19 AFB/1 length	1–29 AFB/1 length
1+	10–99 AFB/1 length or 100 HPF	20–199 AFB/1 length	30–299 AFB/1 length
2+	1–10 AFB/1 HPF on average	5–50 AFB/1 HPF on average	10–100 AFB/1 fieldon average
3+	>10 AFB/1 HPF on average	>50 AFB/1 HPF on average	>100 AFB/1 fieldon average

AFB: Acid Fast Bacilli, HPF: High Power Field.

On a monthly basis, as part of internal quality control (IQC), the study laboratory supervisor read blindly a random sample of 50 to 100% positive ZN smears and 10 to 20% negatives ZN smears. At the end of the study, a random selection of 120 ZN slides were blindly controlled by the mycobacteriology laboratory of the Kenyan Medical Research Institute (KEMRI). As LED-FM slides should be read within 24 hours, as part of the IQC, the laboratory supervisor controlled all LED-FM slides one day (chosen randomly) per week. A random sample of 200 auramine stained smears was blindly read by a 2^nd^ reader, on the same day, to measure the inter-reader reliability of the LED-FM. In addition, a random selection of auramine stained slides were re-read the same day at KEMRI using conventional fluorescence microscope (CFM). Finally, at the end of the study, all auramine slides from a random selection of *patients* were restained with auramine and read blindly by the same reader with LED-FM using two difference examination schemes: A/Under 400 magnification and B/First screening of the slide under 200 magnification and confirmation under 400 magnification (Table 1) [24]. The duration of microscopy needed to identify positive and negative smears using the above schemes was measured. Positive LED-FM slides were not confirmed by restaining and reading with ZN [25]. A questionnaire was developed to investigate the operational aspects of LED-FM.

Patients were asked to produce a 4^th^ sputum specimen in the early morning at home on the 3^rd^ day for *Mycobacterium tuberculosis* (*MTB*) culture used as the reference standard. The 4^th^ specimen was brought to the laboratory on the day patients came to collect microscopy results. Specimens were stored in the fridge and sent twice a week to the KEMRI laboratory where the MTB culture on Lowenstein Jensen (LJ) medium was performed. Specimens were decontaminated using N-acetyl-L-cysteine/sodium hydroxide (NALC/NaOH) followed by neutralization with phosphate buffer, centrifuged and the deposits inoculated on LJ media and incubated at 37°C up to 8 weeks. The cultures were inspected weekly and reported in accordance with the WHO culture grading scale. All positive cultures were confirmed for presence of acid fast bacilli by ZN microscopy. Strain identification was done using temperature growth range, pigment production, resistance to p-nitrobenzoic acid (500 mg/l) and thiopen carboxylic acid hydrazide (2 mg/l) and niacin production [24]. The laboratory participated to external quality assurance programs with the Clinical Sciences Research Centre, Barts London & QM University Hospitals in London (UK) and the Tropical Medical Institute in Antwerp (Belgium).

Patients with at least one positive smear result (≥1 AFB/length), regardless of the microscopy method, were started on treatment. Smear-negative patients were referred to the clinician for further investigation. Patients who were later found to be culture positive were traced and started on treatment.

Data was double entered in an Epidata 3.1 (Epidata, Denmark) database and analysis used the Stata® 9.2 software (College Station, Texas, USA). Sensitivity, specificity and positive and negative likelihood ratio were measured for both ZN and LED-FM methods, using one positive smear (detection of at least 1 AFB per length) out of three and two specimens to define a smear-positive case, respectively. Sensitivity and specificity of both methods were compared using McNemar's test for matched data. The detection yields of ZN and LED-FM microscopy were also calculated per specimen. The inter-reader reliability and reproducibility of reading between the LED-FM and CFM were assessed by the calculation of Kappa coefficients. A Kappa between 0.80 and 1 was considered as perfect agreement.

## Results

**Figure 2:**
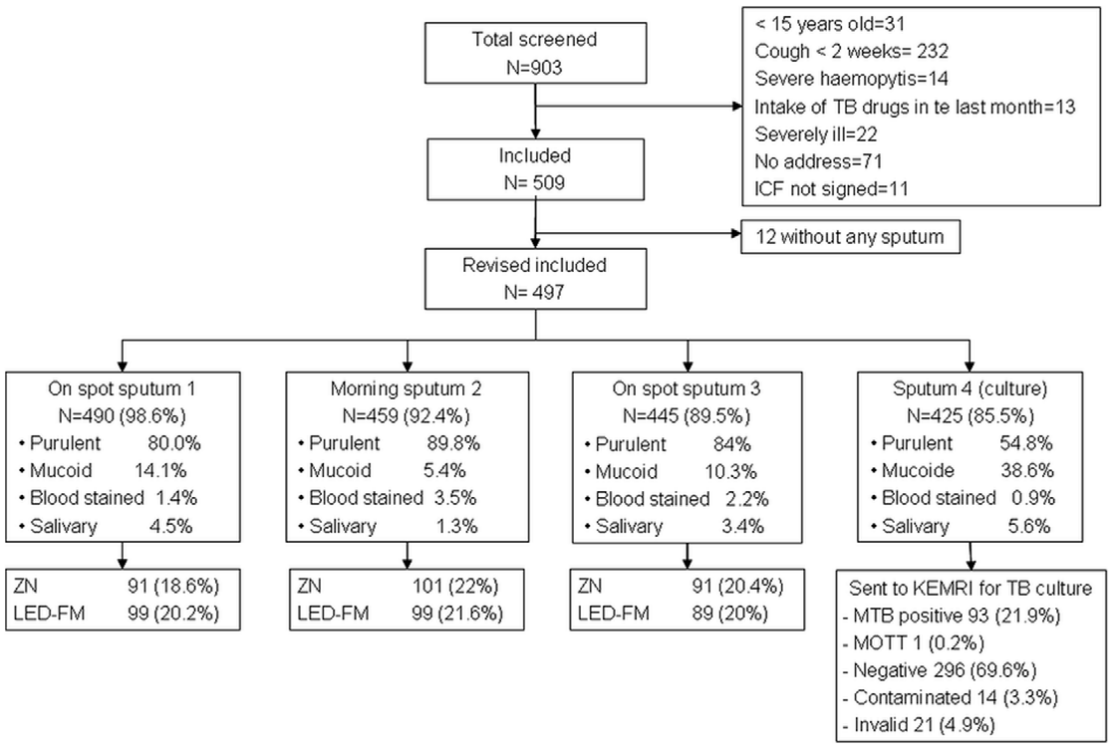
Study profile. ICF: Informed Consent Form, ZN: Ziehl Neelsen, LED-FM: Light Emitting Diode Fluorescence Microscopy; MTB: *Mycobacterium tuberculosis*; MOTT: Mycobacterium Other Than Tuberculosis.

Between May 2008 and May 2009, 509 out of 903 screened patients were included in the study and 497 could produce at least 1 specimen, resulting in 1394 collected specimens (specimen 1, 2 and 3). The study profile and patients' characteristics are presented in Figure 2 and Table 2, respectively. Among 497 patients, 90% were able to produce a set of 3 specimens. Out of 1394 specimens, 1178 (84.5%) were macroscopically purulent or muco-purulent, 140 (10%) mucoid, 33 (2.4%) blood stained and 43 (3.1%) salivary (Figure 2). The morning specimen (2^nd^) was significantly of better quality (purulent or mucopurulent) (412/459) than the 1^st^ (412/459, p<0.001) and 3^rd^ (374/445, p = 0.009) specimens, respectively. Seventy two patients (14.5%) did not submit a 4^th^ specimen for *MTB* culture. The smear positivity rate using ZN microscopy was not different between patients who produced a 4^th^ specimen (22.8%, 97/425) and those who did not (25%, 16/72), p = 0.69. Mycobacterium Other Than Tuberculosis was identified in one patient with positive culture result. The patient smear positive with ZN (scanty result on one specimen) and smear-negative with LED-FM. After exclusion of contaminated and invalid culture results and those with growing mycobacteria other than tuberculosis, 23.9% (93/389) of the patients had a confirmed infection with *MTB* by culture. The 21 invalid cultures resulted from the failure of the decontamination of a batch of specimens due to an error in the preparation of the decontaminant.

**Table 2:** Patients' characteristics.

Characteristics, N = 509	Results
**Mean age** mean (SD)	34.3 (11.6)
**Gender ratio** (M:F)	1.6 (311:198)
**Past TB history** n (%)	105 (20.6)
–Duration since the last TB event in years median (IQR)	4.4 (2.4–7.7)
**Intake of antibiotics in the last 2 weeks n (%)**	169 (33.2)
–Amoxicillin	115 (68)
–Cotrimoxzole	64 (37.9)
–Erythromycine	11 (6.5)
–Teracycline/doycycline	3 (1.8)
–Other	13 (7.7)
**Clinical presentation n (%)**	
–Sputum production	475(93.3)
–History of fever in the last week	395 (77.6)
–Chest pain	402 (78.9)
–Night sweats	399 (78.4)
–Reported weigh loss	409 (80.3)
–Loss of appetite	400 (78.6)
–Haemoptysis	5 (9.8)

Table 3 presents the smear microscopy results per specimen. Using 1 AFB/length cut-off to define a positive smear, there was 283 positive smears (20.3%, 95%CI [18.2–22.5]) with ZN compared to 287 (20.6%, 95%CI [18.5–22.8]) with LED-FM, p = 0.64. Three specimens were ZN positive (1+) and LED-FM negative. Patients were confirmed culture positive in 2 cases and negative in one case. One specimen was LED-FM positive (1+) and ZN negative. Culture was negative. Among 15 discordant results (ZN scanty and LED-FM negative), culture was positive in 6 cases (40%). After exclusion of 5 cases (2 invalid culture results and 3 cases without culture), among 16 scanty LED-FM that were ZN negative, culture was positive in 6 cases (37.5%).

**Table 3:** Smear microscopy results.

LED-FM					
ZN	Negative	Scanty	1+	2+	3+
Negative	1089	21	1	0	0
Scanty	15	42	8	0	0
1+	3	23	44	28	3
2+	0	3	7	47	20
3+	0	0	1	10	29

ZN: Ziehl Neelsen, LED-FM: Light Emitting Diode Fluorescence Microscopy.

Table 4 presents results of the ZN and LED-FM performance when using one positive smear out of three and two (1 and 2) specimens case definition, respectively. Sensitivity and specificity of both microscopy methods were superior or equal to 70% and 95% regardless on the smear positive case definition. There was no difference in sensitivity and specificity between LED-FM and ZN microscopy.

**Table 4:** Performance of smear-microscopy using Ziehl Neelsen and LED-fluorescence microscopy using two smear-positive case definitions.

	ZN		LED-FM		p
	Positive	Negative	Positive	Negative	
**One positive smear out of three smears**					
Positive culture	67	26	68	25	
Negative culture	12	284	9	287	
–Sensitivity, % [95%CI]	72.0 [61.8–81.6]		73.2 [62.9–81.8]		0.32
–Specificity, % [95%CI]	95.9 [93.0–97.9]		96.7 [94.3–98.6]		0.53
–Positive Likelihood ratio	17.8		24.0		
–Negative Likelihood ratio	0.29		0.28		

One positive smear out of the two first smears					
	Positive	Negative	Positive	Negative	
Positive culture	65	28	67	26	
Negative culture	10	286	8	288	
–Sensitivity, % [95%CI]	69.9 [59.5–78.0]		72.0 [61.8–80.6]		0.56
–Specificity, % [95%CI]	96.6 [93.9–98.4]		97.3 [94.7–98.3]		0.37
–Positive Likelihood ratio	20.7		26.7		
–Negative Likelihood ratio	0.31		0.29		

ZN: Ziehl Neelsen, LED-FM: Light Emitting Diode Fluorescence Microscopy.

Inter-reader reliability of LED-FM was measured for 223 smears. There were 7 minor discordances (scanty-negative). Therefore, the positive-negative inter-reader agreement was excellent with kappa value of 0.90 (95%CI [0.83–0.97]). Table 5 presents the results of concordance between LED-FM and CFM. There were 21 smear-positive results with LED-FM that were negative under CFM, compared to only one positive smear result under CFM and negative with LED-FM. The inter-FM reproducibility was good with kappa value of 0.80 (95%CI [0.73–0.88]).

**Table 5:** Results of smear reading using LED-FM and conventional FM (N = 332).

	Conventional FM				
LED-FM	Negative	Scanty	1+	2+	3+
Negative	252	0	**1**	0	0
Scanty	**15**	6	1	1	0
1+	**2**	0	7	9	0
2+	**4**	0	1	11	13
3+	0	0	0	2	9

LED-FM: Light Emitting Diodes Fluorescence microscopy.

The mean reading time (standard deviation, SD) with LED-FM was 1.1 minute (0.38). It was 1 minute (0.52) for smear-positive (N = 287) and 1.1 minute (0.33) for smear-negative slides (N = 1105).

A random sample of 122 patients had all their LED-FM slides re-stained and blindly re-read using two different examination schemes (A/400 or B/200 magnification). There was no difference of smear-positive detection yield between the use of scheme A (22.1%) and scheme B (22.9%), p = 1. The mean reading time (SD) using schemes A and B was 1.15 (0.39) and 1.16 minute (0.45), respectively (p = 1).

There were 5 ZN IQC exercises during the study period. There was no major error out of 110 slides checked and three minor errors (2.7%) [24]. Out of 120 slides randomly selected for external ZN quality control, only 1 scanty result was reported as negative by the controller.

The training of technicians who had great experience in ZN microscopy but none in FM took 2 weeks. There was no need to replace the light-emitting diode despite daily use during the one year study. Technicians declared that the auramine staining was as easy as the ZN staining method to perform and the reading easier with LED-FM. They preferred the FM examination scheme A. They considered the maintenance of the LED system easy. Overall, acceptability was very good.

## Discussion

In 2010 the WHO recommended that conventional FM be replaced by LED-FM in all settings where CFM is used, and that LED microscopy be phased in as an alternative for conventional ZN microscopy [26]. In this large cohort study, LED-FM had the same sensitivity as ZN with similar high specificity. In our study, the specificity was not affected by the use of a very sensitive AFB cut-off (1 AFB/length) to define a positive smear and the high proportion of scanty results (61%) among positive smears.

Smear reading was three times faster with LED-FM (mean time = 1 min) compared to data on conventional ZN microscopy (mean time = 3 min) reported by us in a previous study conducted in same clinic and study population [27]. This is consistent with the 25 to 66% time saving when using FM compared to ZN microscopy reported in two previous studies [13], [17]. Therefore, in programmatic conditions, the introduction of LED-FM would significantly reduce laboratory workloads and possibly allow better quality microscopy to be done with the same human resources compared to ZN microscopy. Indeed, a recent study modelling the potential impact of the new WHO recommendations and the introduction of LED-FM on the TB diagnostic services in Malawi, showed that a substantial increase in smear positive case detection could be obtained using existing human resources and minimal additional equipment [12], [28]. Although LED-FM did not increase sensitivity in this prospective study very good quality smear microscopy was possible, with 30% of the examining time and the same human resource compared to ZN microscopy. This confirms the main predictions of the model. The LED-FM would be particularly adapted to high HIV prevalence countries, which face crisis of human resource for health [29].

The inter-reader reproducibility of LED-FM was excellent (kappa = 0.9). Although very high, the reproducibility of smear reading between CFM and LED-FM was slightly lower (k = 0.8), especially with the scanty smears. This might be explained by the fact that the CFM and LED-FM readings were done by two different microscopists (on site and at KEMRI) and that KEMRI microscopists were used to the old grading system where smears with 1–3 AFB in a recommended field were considered as negative [30]. It could be also explained by a better detection yield of the LED-FM technology or by a suboptimal CFM if the mercury vapour lamp bulb was approaching or had exceeded its working-life [18].

Two different FM examination schemes were compared in this study. Pre-screening of the smear under X200 magnification with confirmation by X400 didn't increase sensitivity compared to direct screening under X400 magnification but more surprisingly, there was no reduction of time to read a smear when using the X200 magnification scheme. Technicians reported that it was less easy to detect fluorescence under X200 compared to X400 magnification and that it required more focus time as 2 different objectives had to be used in case of AFB seen under X200. Also, a recent report showed a better correlation of smear positive results between different LED systems when using 400 magnification compared to 200 magnification [31].

Regarding feasibility aspects, this study confirmed the very good acceptability of the LED-FM by technicians, the long working-life of the diodes in the conditions of a peripheral laboratory and the possibility to use LED-FM without a dark room. These are all very important considerations in scaling-up LED-FM in national TB programs with limited resources. In preparation for this study, the training of TB microscopists who had no previous experience with FM was straightforward.

This study has some limitations. The absence of an increase in sensitivity might be explained by the great experience of study technicians with the ZN method and the fact they never used FM before being trained for the study. Because it was not possible to offer HIV testing and counselling to all TB suspects, the analysis couldn't be stratified per HIV status. For methodological reasons, the culture reference standard was not processed on all specimens used for microscopy but on a different specimen. Not all patients were able to produce a 4^th^ specimen for culture and among those who did, the macroscopic appearance of the 4^th^ specimen was of poorer quality than those specimens submitted for microscopy. We can't really explain the poor quality of the culture specimen that was an early morning specimen, expected to be of good quality. It might be possible that patients were fatigued with the process of producing more specimens. This could have been avoided with specimen homogenisation before splitting samples for microscopy and culture, but such practice would have not reflected what is done in routine conditions, where microscopy is performed directly on fresh specimen. Nevertheless, although this might have slightly biased the performance of the microscopy methods, it is unlikely that it affected the difference of sensitivity between LED-FM and ZN microscopy. Indeed, the comparison of detection yields and sensitivities gave similar results.

In conclusion, this study shows that LED-FM might not always increase sensitivity compared to ZN microscopy. Nonetheless the faster reading of LED-FM smears combined with excellent inter-reader reproducibility, very good acceptability and ease of use, would support its introduction in peripheral laboratories in resource-poor settings. The development and validation of adequate and sustainable external quality assessment systems for LED-FM still remain a pre-condition for the scale-up of LED-FM [28].
